# The Pestivirus N Terminal Protease N^pro^ Redistributes to Mitochondria and Peroxisomes Suggesting New Sites for Regulation of IRF3 by N^pro^

**DOI:** 10.1371/journal.pone.0088838

**Published:** 2014-02-14

**Authors:** Matthew Jefferson, Matthew Whelband, Irina Mohorianu, Penny P. Powell

**Affiliations:** Biomedical Research Centre, Norwich Medical School and Biological Sciences, University of East Anglia, Norwich, United Kingdom; McMaster University, Canada

## Abstract

The N-terminal protease of pestiviruses, N^pro^ is a unique viral protein, both because it is a distinct autoprotease that cleaves itself from the following polyprotein chain, and also because it binds and inactivates IRF3, a central regulator of interferon production. An important question remains the role of N^pro^ in the inhibition of apoptosis. In this study, apoptotic signals induced by staurosporine, interferon, double stranded RNA, sodium arsenate and hydrogen peroxide were inhibited by expression of wild type N^pro^, but not by mutant protein N^pro^ C112R, which we show is less efficient at promoting degradation of IRF3, and led to the conclusion that N^pro^ inhibits the stress-induced intrinsic mitochondrial pathway through inhibition of IRF3-dependent Bax activation. Both expression of N^pro^ and infection with Bovine Viral Diarrhea Virus (BVDV) prevented Bax redistribution and mitochondrial fragmentation. Given the role played by signaling platforms during IRF3 activation, we have studied the subcellular distribution of N^pro^ and we show that, in common with many other viral proteins, N^pro^ targets mitochondria to inhibit apoptosis in response to cell stress. N^pro^ itself not only relocated to mitochondria but in addition, both N^pro^ and IRF3 associated with peroxisomes, with over 85% of N^pro^ puncta co-distributing with PMP70, a marker for peroxisomes. In addition, peroxisomes containing N^pro^ and IRF3 associated with ubiquitin. IRF3 was degraded, whereas N^pro^ accumulated in response to cell stress. These results implicate mitochondria and peroxisomes as new sites for IRF3 regulation by N^pro^, and highlight the role of these organelles in the anti-viral pathway.

## Introduction

Viruses employ many strategies to establish persistent infection of their host. Most involve inhibition of innate immune responses, including inhibition of Type I interferon and inhibition of apoptosis [1], [2]. Pestiviruses, which include classical swine fever virus (CSFV) and bovine viral diarrhea virus (BVDV) generate persistent infections as both viruses block interferon synthesis and apoptosis [3]. CSFV and BVDV are members of the Flavivirus family, with a single positive-stranded RNA genome of 12 kb which encodes a single large polyprotein. N^pro^ is a unique autoprotease, located at the N-terminus which cleaves itself from the polyprotein soon after translation on ribosomes [4]. Our previous work has shown that this single viral protein expressed alone in cells can block Type 1 interferon production by promoting the loss of IRF3 [5], to which it binds directly through a zinc binding TRASH motif [6], leading to IRF3 degradation in proteasomes [7]. Both these properties of N^pro^ have generated useful biological tools; the autoprotease activity has been used to enhance refolding and expression of fused peptides and proteins during bacterial protein expression [8] and the direct antagonism of IRF3 has been used to generate cell lines deficient in IRF3 to block antiviral responses to study viral replication [9].

Recent work has linked the central role played by IRF3 in signaling both interferon production and apoptosis [10], however little is known about the IRF3 signaling pathways targeted by N^pro^ to inhibit the apoptotic pathway, important in generating persistent viral infections. IRF3 is activated by dsRNA interaction with Toll-like receptors and the RNA helicases family (RLRs), including Rig-I and mda5. Binding of viral dsRNA to RNA helicases results in the activation of a CARD domain in Rig-I and mda5, allowing them to associate with the mitochondrial adaptor protein MAVS/IPS-1 [11], [12]. MAVS acts as a platform to recruit several cytosolic protein kinase complexes to mitochondria, including I kappa B kinase and tank binding kinase-1, which phosphorylate IRF3, resulting in IRF3 translocation to the nucleus to bind to interferon-sensitive response elements (ISRE) and turn on interferon stimulated genes (ISGs) [13]. In addition, IRF3 translocates to mitochondria through its interaction with the pro-apoptotic protein Bax to activate the intrinsic apoptotic pathway [14]. This and the fact that MAVS is located predominantly on the outer mitochondrial membrane have highlighted the importance of mitochondria in host innate immune signaling [15]. Interestingly, MAVS has also been found to localize to peroxisomes, [16] a cytosolic organelle important in lipid oxidation, which under normal physiological conditions proliferates by fission [17]. However, recent evidence has described peroxisomes originating from the endoplasmic reticulum and exchanging proteins with mitochondria, forming an ER sub-compartment called the pre-peroxisomal reticulum [18], but this is a matter of considerable debate [17]. The peroxisome pool of MAVS was shown to induce an immediate antiviral response of interferon stimulated genes, such as viperin, ISG15 and ISG56, but not type I interferon itself [16].

Given that the mechanism and site of action of N^pro^ is poorly understood, we investigated its cellular distribution when expressed alone in cells following induction of cellular stress, since virus entry is known to activate the cellular stress pathway to induce the innate response [19]. Initially, N^pro^ relocated to tubular mitochondria, and the pro-apoptotic protein Bax remained cytoplasmic with the subsequent inhibition of apoptosis. Mutations in N^pro^ that prevented binding and loss of IRF3 did not inhibit stress-induced apoptosis, and Bax redistributed to spherical, fragmented mitochondria, revealing the inhibition involved the inactivation of Bax by the loss of IRF3. Interestingly, both N^pro^ and IRF3 were recruited to mitochondria and also to spherical structures identified as peroxisomes, and led to an increased accumulation of ubiquitin associated with peroxisomes. The results show the prevention of apoptosis by N^pro^ depends on its capacity to bind and eliminate IRF3, supporting a direct role for IRF3 in initiating the intrinsic mitochondrial pathway of apoptosis. N^pro^ redistributed to cellular platforms important for IRF3 signaling, implicating mitochondria and peroxisomes as new sites for IRF3 regulation by N^pro^.

## Materials and Methods

### Cells and Reagents

Mouse embryonic fibroblast (MEFs), HeLa and Mardin-Darby Bovine Kidney (MDBK) cells were obtained from European Collection of Cell Cultures (ECACC). MEF and HeLa cells were maintained in DMEM with GlutaMAX with 10% fetal calf serum. MDBK cells were grown in MEM and 10% BVDV- free media with Glutamax (Invitrogen). Cells were seeded on coverslips in 24-well plates and infected with BVDV Kyle non-cytopathic strain of BVDV (from J. Brownlie, Royal Vet College, South Mimms UK). After absorption of virus for 1 hour, cells were fed with fresh media and incubated overnight at 37 C before treatment or transfection with DNA. Reagents were from Sigma unless other stated. Cells were treated with sodium arsenate (NaA) at 100 uM, staurosporine at 20 nM, hydrogen peroxide at 200 µM. Interferon alpha (PBL assay science) was used at 100 U/ml, and poly I:C (Roche) was transfected into cells at 100 µg/ml using lipofectamine (Invitrogen).

### Plasmids and Transfections

N^pro^cherry was generated from pcDNA3 N^pro^ [5] and cloned in-frame into pcDNA3mCherry (Invitrogen). N^pro^ mCherry point mutants C112R and D136N were created using the Quick Change kit (Stratagene). Plasmids were transfected into cells with jetPRIME (Polyplus) and cell lines stably expressing N^pro^ mCherry or mutant proteins were selected with G418 (1 mg/ml, Invitrogen). Plasmids encoding tBid-GFP [20] and BAX -GFP (from Stephen Tait, Beatson Institute of Cancer Sciences, University of Glasgow) and IRF3-GFP (from John Hiscott, MacGill University, Canada) were transfected into cells using Fugene HD (Roche). MDBK cells were transfected with plasmids encoding Bax-GFP using jetPRIME (Polyplus) according to the manufacturer’s instructions.

### Protein Extraction and Western Blotting

Protein was extracted with M-PER (Pierce) for 30 minutes on ice in the presence of HALT protease inhibitor cocktail (Pierce). Protein concentrations were determined using the BCA protein assay kit (Pierce) according to the manufacturer's protocols. Equal amount of proteins were separated on 4–12% SDS-PAGE gradient gels, and analyzed by blotting onto Immobilon PVDF membranes using antibodies to IRF3 (Abcam Ab25950) and antibodies to N^pro^, produced in rabbits immunized with synthetic peptides for N^pro^ conjugated to OVA and KLH as described previously [5]. Equal protein loading was confirmed by staining with anti-beta-actin monoclonal antibody AC-15 (Sigma A5441). Primary antibodies were detected using IRDye-labelled secondary antibodies (Li-Cor biosciences) at 1∶5000 dilution or HRP conjugated secondary antibodies at 1∶500 (Jackson Immunoresearch, Europe). Proteins were visualised either by Odyssey infrared system, or enhanced chemi-luminescence activity. Membranes were stripped and re blotted using Re-blot plus strong solution (10×) (Millipore, 2504). Bands were quantitated using the Odyssey software or Scion image software http://scion-image.software.informer.com.

### Immunofluorescence and Image Analysis

Cells were fixed in 4% paraformaldehyde and permeabilised with Triton-X100. Cells were stained in 30% goat-gelatin buffer with anti-ubiquitin monoclonal antibody FK2 (Enzo PW8810) or anti-rabbit peroxisome antibody PMP70 (Sigma P0497). Alexa fluorescent conjugated anti- rabbit and anti-mouse secondary antibodies were from Invitrogen and donkey anti-mouse Cy5 was from Jackson Immunoresearch, Europe. For BVDV staining, MDBK cells were fixed in ice cold 80% acetone and BVDV glycoproteins were stained with bovine hyperimmune serum V182 against BVDV (a gift from J. Brownlie, Royal Vet College, South Mimms) and visualized with a secondary anti-cow Cy 3 (Jackson Immunoresearch, Europe). MitoTracker CMXRos (Molecular Probes, Invitrogen) was used at a final concentration of 400 nM and applied to live cells 30 minutes before fixation. DNA was stained with DAPI (Sigma D9542) and mounted with Fluoromount G (Southern Biotech). The fixed cell images were obtained at ×63 magnification on a Zeiss Axioplan 2 or a Zeiss laser-scanning confocal microscope LSM510. Images were analysed using the Axioplan software version 4.8. For pixel density analysis, images were analyzed using Imaris ×64 7.2.3 software (Bitplane Software Incorporated). Punctate structures within the images were identified using the “spots” function, and larger structures, such as nuclei, were identified using the “surfaces” function within the software. These object layers were used to create the rendered images and to assimilate data concerning vesicle number.

### Caspase 3/7 Apoptosis Assay

Following cell treatment, caspase activity was measured in cell lysates using the Apo-ONE Caspase-3/7 Assay (Promega) in 96-well plates with the addition of the fluorescent substrate Z-DEVD. Fluorescence was measured using Envision plate-reader (PerkinElmer). Fold induction of enzyme activity was obtained by dividing the activity in the treated samples by the activity obtained in the untreated cells.

## Results

### Inhibition of Caspase-dependent Apoptosis by N^pro^ Correlates with Degradation of IRF3

**Figure 1:**
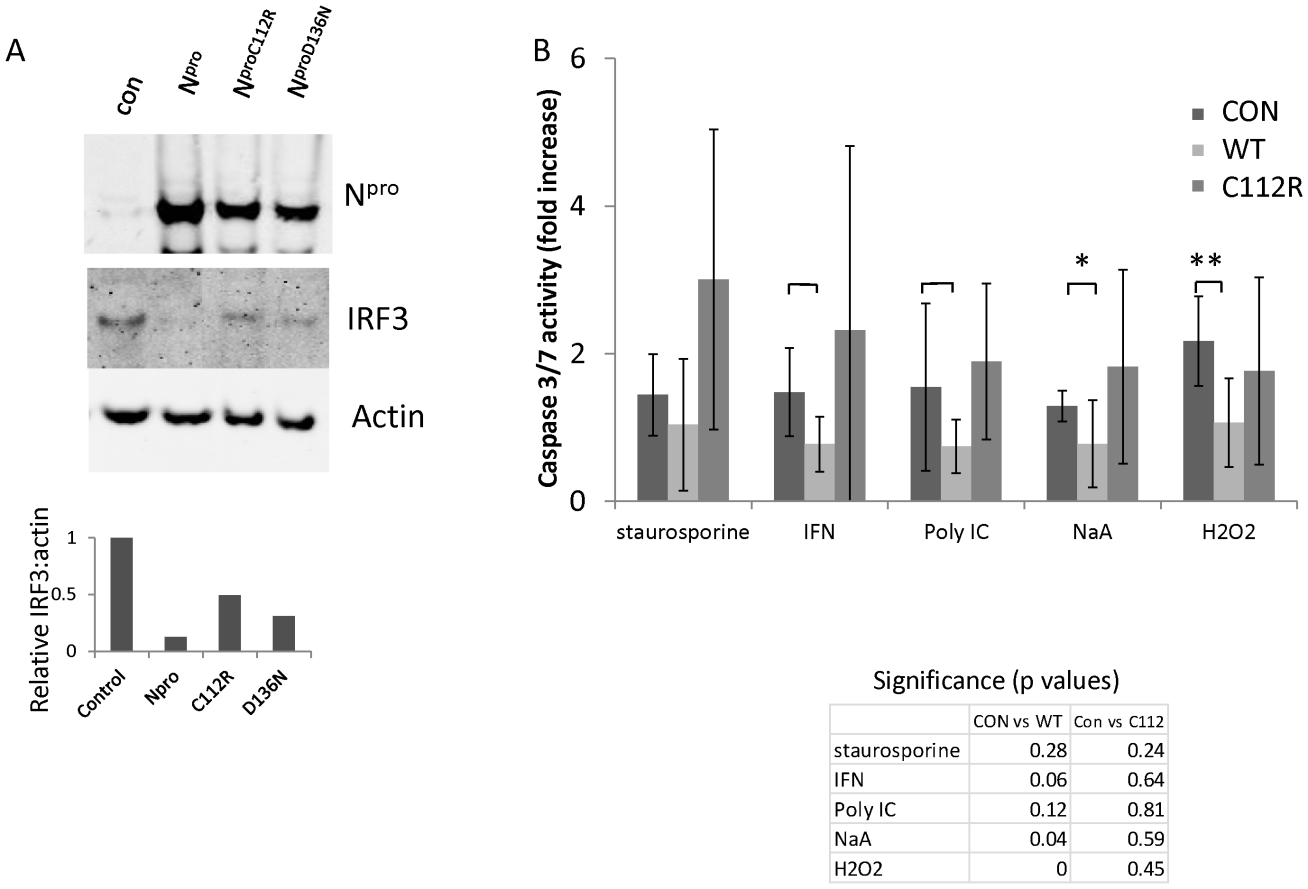
Inhibition of apoptosis by N^pro^ correlates with degradation of IRF3. (A). Western blotting of lysates from MEF cell expressing N^pro^ cherry with antibodies against N^pro^, IRF3 and actin shows loss of IRF3 from cells expressing N^pro^. Lane 1: Control MEF cells not expressing plasmid. Lane 2: MEF cells stably expressing N^pro^ mcherry. Lane 3 : MEF cells expressing N^pro^ C112R mcherry mutant. Lane 4: MEF cells expressing N^pro^ D136N cherry mutant. Graph shows relative intensities of IRF3 compared to actin for the image scanned with Scion image software http://scion-image.software.informer.com (representative of Western blot repeated three times for each lane). (B). N^pro^ but not N^pro^ C112R inhibits caspase 3/7 activity. MEF cells stably expressing wild type N^pro^ or mutant N^pro^ C112R mcherry were treated with staurosporine, interferon, dsRNA (poly I:C), sodium arsenate (NaA) or hydrogen peroxide (H_2_O_2_) for 4 hrs. Fold increase in caspase activity was normalized to negative (untreated cells) for each cell line and analyzed with a two-sided t-test with unequal variance relative to the untransfected cells for each cell line, and significance values are: bars marginally significant (0.05–0.1), * significant (0.01–0.05), ** highly significant (0–0.01) (n = 5 to 8). The table shows significance of drug treatment relative to control treated cells for cells expressing N^pro^ WT or N^pro^C112R and analysis was conducted using similar tests as described above.

As we have previously shown that virus or N^pro^ expressed alone in cells can promote the degradation of IRF3 [5], we analyzed the levels of IRF3 in cell lines stably expressing wild type N^pro^ fused to m-cherry, or cells expressing N^pro^ carrying mutations in cysteine 112 or aspartic acid 136 residues. We tested these point mutations because previous work has implicated these individual mutations in the loss of IRF3 when introduced into the virus, abolishing IRF3 binding and interferon inhibition [21]. A representative Western blot in Figure 1A shows that IRF3 was undetectable in cells expressing wild type N^pro^ and detectable, but reduced compared to normal, in cells expressing N^pro^ mutations C112R and D136N. Quantitation of IRF3 bands against actin intensity showed expression of N^pro^ C112R or Npro D136N led to approximately 50% of the IRF3 levels seen in control cells (Figure 1A graph), indicating that there was less efficient degradation of IRF3 compared to cells expressing wild type N^pro^ but the mutants were not completely inactive. The two residues at 112 and 136 have been shown to be part of a site for co-ordination of zinc; however proteins where these residues were mutated were found to still contain up to 26% of control levels of zinc [6].

The ability of N^pro^, or mutated N^pro^ proteins to protect cells from apoptosis after challenge for 4 hours with inducers of caspase-dependent apoptosis is shown in Figure 1B. Each chemical stimulates a different pathway, but each leads to the activation of caspase 3. Caspase 3/7 activity was measured after 4 hours using luminogenic substrate and results were compared to untreated cells which were normalized to 1. Cells expressing wild type N^pro^ had reduced caspase 3/7 activation by staurosporine, interferon, poly IC, sodium arsenate, and H_2_O_2_ compared to control cells not expressing N^pro^. Cells expressing N^pro^ mutated at positions C112R significantly reversed inhibition of caspase activation when compared to cells expressing wild type N^pro^. The significance values for caspase activation by each drug treatment for cells expressing viral protein compared to the drug treatment of control cells are shown in the panel below the graph (Figure 1B). Treatment with sodium arsenate and hydrogen peroxide gave the most significant values for inhibition of apoptosis by N^pro^. Both chemicals trigger apoptosis via a reduction in mitochondrial membrane potential, cytochrome c release, and caspase activation. Sodium arsenate is also an inducer of the cell stress pathway and we chose this chemical to look at the role of N^pro^ in inhibition of apoptosis, both because mitochondria play a central role in IRF3 signalling and because virus infection activates the cell stress pathway [19].

### N^pro^ Prevents Fragmentation of Mitochondria following Cell Stress

**Figure 2:**
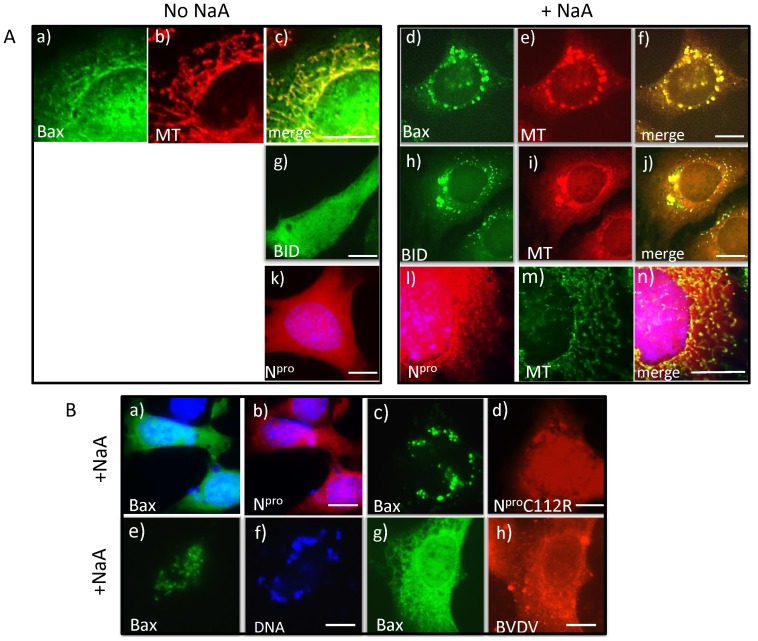
N^pro^ is recruited to mitochondria after cell stress and protects from apoptosis. (A). MEF cells were transfected with Bax-GFP alone (a–f) or tBid-GFP alone (g–j) or N^pro^ cherry alone (k–n) and either untreated (No NaA, left panel) or treated with sodium arsenate for 4 hours (+NaA, right panel). Cells were incubated with Mitotracker (MT) CM Ros (b,e,i) or Green FM (m) for 30 mins before fixation. (B). Protection from apoptosis induced by sodium arsenate requires IRF3. MEF cells co-expresssing Bax-GFP and either wild type N^pro^-cherry (a–b) or mutant N^pro^ C112R-cherry (c–d) were treated with sodium arsenate (+NaA). MDBK primary cells, transfected with Bax GFP (e–f) alone or infected with BVDV overnight and then transfected with Bax GFP (g–h), were treated with sodium arsenate (+NaA) for 4 hours. BVDV was detected with bovine hyperimmune serum V182 and detected with Cy3 rabbit anti bovine antibody (red) and DNA was stained with DAPI.

It has been shown that apoptosis may be induced during viral infection by IRF3-mediated activation of Bax [14]. We asked whether the mechanism of apoptosis inhibition by N^pro^ involved the intrinsic mitochondrial pathway which is dependent on IRF3 interaction with Bax, by investigating the location of Bax-GFP, tBid-GFP and N^pro^ before and after treatment with sodium arsenate, an inducer of cell stress and apoptosis as shown in Figure 1B above. In normal cells, Bax generally distributed to cytoplasm and a fraction to mitochondria (Figure 2A: a–c), loosely bound to the outer mitochondrial membrane [22], [23]. tBid and N^pro^ localised to the cytoplasm and nucleus (Figure 2A: g and k). Following treatment with sodium arsenate, Bax and tBid redistributed to small, punctate perinuclear structures that co-localised with mitochondria identified with Mitotracker (MT) (Figure 2A: d-f and h-j respectively). This mitochondrial fragmentation has been previously shown after cell stress, and it demonstrates that Bax and Bid remain associated following fragmentation, and cells die by apoptosis [23]. In cells expressing N^pro^ alone following treatment with sodium arsenate, N^pro^ also co-localised with mitochondria (Fig. 2A: l–n), but in contrast to the spherical, perinuclear puncta seen in cells expressing Bax or tBid alone, the mitochondria remained tubular and spread throughout the cytoplasm. N^pro^ expression stabilized the tubular mitochondrial network and the cells were protected from apoptosis. In addition, this shows that since levels of IRF3 are depleted (Figure 1A), N^pro^ association with mitochondria is independent of its binding to IRF3. In the next experiment, when both Bax-GFP and N^pro^-cherry were expressed together, Bax distribution remained unchanged after addition of sodium arsenate in the cytoplasm and a fraction on mitochondria (Fig. 2B: a–b), demonstrating that when cells are stressed, in the presence of N^pro^ and the absence of IRF3, cytoplasmic Bax does not redistribute to spherical fragmented mitochondria and cells are protected from apoptosis. However, in cells expressing the N^pro^ C112R mutant unable to bind and target IRF3 for degradation or protect cells completely against apoptosis (Figure 1), Bax located to spherical perinuclear punctae (Fig. 2B: c), and the cells died. N^pro^ redistributed to mitochondria and cytoplasmic puncta (Figure 2B: b and d), and the nature of these puncta were further investigated in the next series of experiments.

To investigate Bax localization during virus infection, the experiment was repeated using primary Madin-Darby bovine kidney cells (MDBK cells) infected with bovine viral diarrhea virus (BVDV) Kyle, a non-cytopathic strain. In control MDBK cells treated with sodium arsenate for 4 hours, Bax GFP relocated to spherical perinuclear punctae and cells died by apoptosis, seen by chromatin condensation (Fig. 2B:e–f). In contrast, in MDBK cells infected with BVDV for 24 hours, Bax showed a cytoplasmic and partly mitochondrial distribution (Fig. 2B: g–h) and cells were protected from apoptosis.

Taken together, the results suggest that following induction of cell stress, as occurs during viral infection, N^pro^ redistributes and associates with mitochondria, not only to manipulate the innate immune pathway but also to promote the survival of cells. Both of these effects are through its interaction with IRF3. That the loss of the pro-apoptotic effect of Bax is an indirect effect of N^pro^, due to the loss of IRF3, is supported by the fact that following expression of mutant N^pro^, which does not cause degradation of IRF3, Bax relocates to spherical fragmented mitochondria. It has been found that IRF3 binding to Bax is required for a conformational change in Bax to take place to bind to mitochondria and regulate the permeability of the outer mitochondrial membrane (14). Thus both virus and the single viral protein, N^pro^ inhibit apoptosis through targeting of the Bax mitochondrial apoptotic pathway.

### N^pro^ Rapidly Redistributes with IRF3 to Mitochondria and Peroxisomes

**Figure 3:**
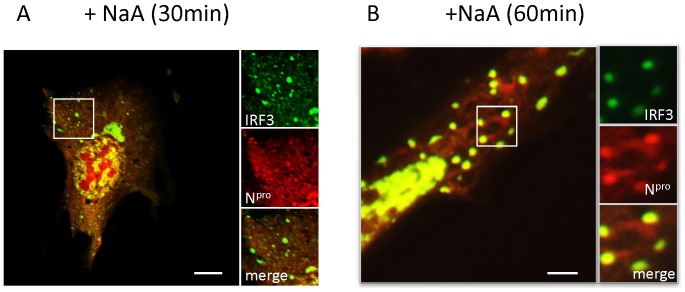
N^pro^ rapidly redistributes with IRF3 to mitochondria and cytoplasmic puncta following induction of stress. (A). MEF cells co-expressing N^pro^ -cherry (red) and IRF3-GFP (green) were treated with sodium arsenate (+NaA) for 30 minutes and examined by confocal microscopy. (B). Treatment of MEF cells co-expressing N^pro^-cherry and IRF3-GFP for 60 minutes.

To investigate the cellular localisation of N^pro^ expressed together with IRF3 before the disappearance of IRF3, (as would be the case immediately after infection), cells stably expressing N^pro^ -cherry were transfected with IRF3-GFP and visualized by confocal microscopy over a time course of treatment (Figure 3). At 30 minutes post-stimulation, N^pro^ was associated with tubular structures and round punctate structures co-localizing with Mitotracker (Figure 3A). Mitochondria were seen as tubular and punctate structures. IRF3 was seen in discrete punctate structures associated with N^pro^ (Figure 3A). After 60 mins, N^pro^ remained associated with tubular mitochondria but the size of N^pro^ cytoplasmic puncta increased. IRF3 was almost exclusively co-localised with N^pro^ in larger ovoid puncta in the cytoplasm, or in the nucleus (Figure 3B).

**Figure 4:**
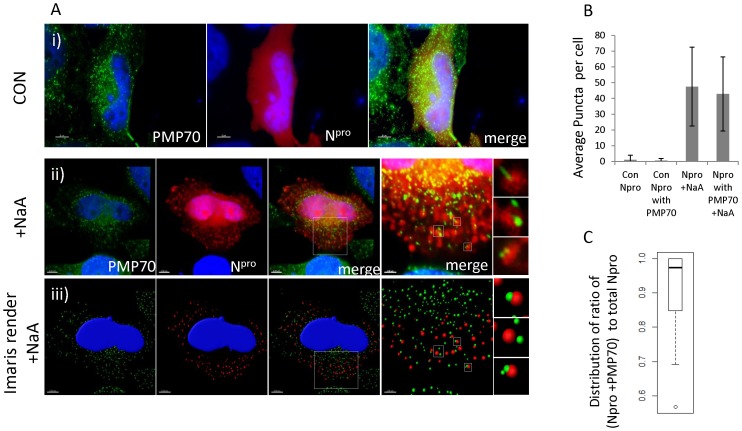
N^pro^ co-distributes with peroxisomes marker PMP70. (A). Peroxisomes were localized by immunostaining with anti-peroxisome marker PMP70 in MEF cells expressing N^pro^ cherry i) control cells (CON) ii) treatment with sodium arsenate (+NaA) for 4 hours iii) Digital rendering using Imaris software is shown for the images in ii) (scale bars are 5 um). (B). Statistical analysis of average number of N^pro^ puncta per cell and average number co-distributing with PMP70 after sodium arsenate treatment for 4 hours. (C). Boxplot: distribution of the fraction of N^pro^ distributing with PMP70 compare to total number of N^pro^ granules. A Welch two sample t-test was applied, two-sided, not paired, (assuming that the variances are unequal) and showing t = 0.5867, df = 25.712, p-value = 0.5625 with the lower end of the box = 25% of the data, bold line in the middle = median, Upper end of the box = 75%, lower whisker = 5%.

As a central regulator of interferon signaling, IRF3 and MAVS have been reported to locate to both mitochondria and to peroxisomes in response to cell stress [16] and since this implicates peroxisomes in anti-viral signaling, we investigated whether the IRF3 and N^pro^ puncta detected in Figure 3 were related to peroxisomes. In control cells, peroxisomes were seen by immunostaining with PMP70 as abundant small punctate cytoplasmic spheres and when N^pro^cherry was co-expressed there was no obvious effect on numbers or sizes of peroxisomes (Figure 4Ai). Following treatment with sodium arsenate, N^pro^ accumulated in cytoplasmic puncta as before and many of these puncta co-distributed with peroxisomes (Figure 4Aii). A statistical analysis was performed using Imaris software and the digital rendering of pixel densities in fluorescent punctae for the same cells is shown below (Figure 4Aiii) Figure 4B shows the average number of N^pro^ granules per cell treated with sodium arsenate for 4 hours, and the average number of N^pro^ granules associated with PMP70 per cell. The box plot in Figure 4C is a statistical analysis of the number of puncta containing PMP 70 which also contained Npro (Figure 4C; box plot) and shows a distribution of 75% in the upper end of the box give a ratio of 0.85 puncta with both N^pro^ and PMP70 compared to the total number of N^pro^ granules. This shows that most of the N^pro^ granules associate with peroxisomes 4 hours after treatment with sodium arsenate.

### N^pro^ and IRF3 Co-distribute to Cytoplasmic Peroxisomes Associated with Ubiquitin

**Figure 5:**
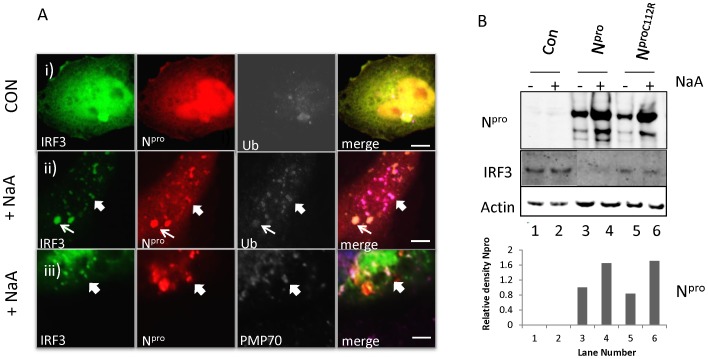
N^pro^ and IRF3 co-distribute to cytoplasmic peroxisomes containing ubiquitin. (A). Cells expressing IRF3 GFP and N^pro^ cherry i) Control cells (CON) were stained for ubiquitin with monoclonal antibody FK2 visualized with anti-mouse Cy5. ii) Cell treated with sodium arsenate (+NaA) for 60 mins and ubiquitin was stained with monoclonal antibody FK2 and visualized with anti-mouse Cy5. iii) Cells treated with sodium arsenate (+NaA) for 60 mins and peroxisomes were stained with anti-rabbit PMP70 and visualized with anti- rabbit Cy5. (B). N^pro^ is stabilized and IRF3 degraded following sodium arsenate treatment. Control cells, cells expressing N^pro^ and cells expressing N^pro^ C112R mutant were untreated (−) or treated with sodium arsenate for 4 hours (+NaA). Lysates were probed by Western blot for N^pro^, IRF3 and actin. Actin is shown for equal lane loading and bands were scanned and normalize with actin for N^pro^ levels in treated and untreated cells (bottom graph).

Since binding to N^pro^ results in increased degradation of IRF3 by the proteasome [7], we looked for the site of ubiquitin accumulation in cells expressing N^pro^ following stress. In control cells co-expressing N^pro^ and IRF3-GFP together and immunostained for polyubiquitin (Figure 5Ai), IRF3 and N^pro^ were seen throughout the cytoplasm and at a perinuclear site which contained a small amount of ubiquitin. Sodium arsenate rapidly induced the formation of small (block arrow) and large (arrow) N^pro^ puncta positive for IRF3 and both forms localized with ubiquitin (Fig. 5Aii). The small punctate structures were confirmed as peroxisomes using an anti-peroxisome antibody PMP70 (Fig. 5Aiii). The large puncta remain unidentified, but are not peroxisomes. Mutant N^pro^ also redistributed to these puncta (not shown), indicating that redistribution was independent of binding to IRF3, however we cannot exclude that the redistribution of IRF3 to peroxisomes may depend on its binding to N^pro^. These results indicate that IRF3 and N^pro^ redistribute together to peroxisomes and larger cytoplasmic stress granules where ubiquitin accumulates. Although IRF3 and N^pro^ movement to the peroxisomes correlated with accumulation of ubiquitin, we were unable to show by Western blot whether IRF3-specific ubiquitination occurred in this subcellular organelle or if the rate of IRF3 degradation was increased following sodium arsenate treatment (Figure 5B, middle panel). Interestingly, however, when we looked at total levels of N^pro^ in cells following cell stress compared to unstressed cells, we found that N^pro^ protein levels increased (Fig. 5B: N^pro^, lanes 3 and 4). Also levels of mutant N^pro^ C112R increased, showing this accumulation was independent of binding to IRF3 (Fig. 5B lanes 5–6). These results demonstrate that peroxisomes are one possible site of IRF3 ubiquitination.

## Discussion

Anti-viral signaling mediated by IRF3 leads to apoptosis and increased interferon production. Our previous work has shown that N^pro^ inhibits interferon signaling by accelerating the degradation of IRF3 during virus infection or when expressed alone in porcine cell lines [5]. We show here that these intrinsic properties of the protein are transferable to other species since they are also exhibited when expressed in mouse and human cells. The mutation of residues involved in the co-ordination of zinc led to a partial but not complete reversal of IRF3 degradation. N^pro^ contains a C-terminal TRASH motif [24] consisting of the amino acids C112-C134-D136-C138 which act together to co-ordinate a zinc atom. Mutation of a single amino acid may not completely abolish zinc and therefore IRF3 binding, because D136N mutation still contained 26% of the wild type zinc levels [6], and mutation of two or more residues is necessary to completely inactivate the motif. In this study, various apoptotic signals induced by staurosporine, poly I:C, interferon, sodium arsenate and hydrogen peroxide were inhibited by wild type N^pro^, but not by the mutant protein, suggesting that the mechanism of apoptosis inhibition is also through IRF3 and that levels of IRF3 in cells expressing mutant N^pro^ were sufficient to promote apoptosis. The subcellular localization of N^pro^ following induction of cellular stress showed that N^pro^ associated with tubular mitochondria, and its expression prevented mitochondrial fragmentation and Bax redistribution, thus inhibiting the Bax-dependent intrinsic apoptotic pathway. Since mutant N^pro^ unable to bind IRF3 did not protect against apoptosis and mitochondria fragmention, it suggests that inhibition of apoptosis is a consequence of reduced IRF3, and not a direct action of N^pro^, although it does not rule out that this mutation interferes with a direct action of N^pro^ on mitochondrial fission. Our work supports the recent finding that IRF3-dependent activation of Bax is an important part of the intrinsic mitochondrial apoptotic pathway [14], where the BH3 domain of IRF3 binds to cytosolic Bax and induces translocation of Bax to mitochondria to activate apoptosis in response to Sendai virus and double stranded RNA. Here we show that sodium arsenate induction of apoptosis and stress is also dependent on IRF3 interaction with Bax since the pathway is compromised when IRF3 is degraded by N^pro^. However, the situation may be more complex since transcription of genes regulated by IRF3 and involved in apoptosis will also be decreased. Our results show that Bax redistribution was inhibited in the same manner during BVDV infection. Many large DNA viruses target the mitochondrial pathway, encoding bcl2 homologues to manipulate apoptosis [25], and our work shows smaller RNA virus with limited coding sequence, manipulate both apoptosis and innate immune pathways through targeting mitochondria as an important sites of IRF3 signaling. N^pro^ was localized both to the mitochondrial tubular network, as well as to punctate structures containing the peroxisome marker PMP70 that were in close proximity to mitochondria, but excluded the mitochondrial marker Mitotracker. The peroxisome is emerging as an important platform for regulation of innate immune responses to viral infection. Several other important host anti-viral proteins which localize to peroxisomes include MAVS [26], LSm14A, a sensor for both viral RNA and DNA [27] and also a fraction of Rig-I [27]. The pool of MAVS located to peroxisomes activates the early rapid and transient expression of ISGs that is independent of interferon production [16]. In addition, MAVS recuitment to peroxisomes modulates the NF-kB signaling pathway [28], and notably, we have previously shown that N^pro^ interacts with I-kBα, the inhibitor of NF-kB [29]. Taken together, the peroxisome platform is an important site of N^pro^ function in inhibiting the anti-viral pathway. Viral proteins that have been shown to localize to peroxisomes [30] include VP4 spike protein of rotovirus [31], and interestingly, NS1 the non-structural protein of influenza virus, which binds to a multifunctional peroxisomal enzyme [32]. In common with N^pro^ when transfected into cells, NS-1 showed a punctate immunofluorescence pattern characteristic of peroxisomes and it can also inhibit interferon production [32]. Significantly, there was a rapid relocation of N^pro^ to peroxisomes containing ubiquitin. The co-localisation of IRF3 and N^pro^ with ubiquitin in peroxisomes suggests a link between IRF3 binding to N^pro^ and rapid IRF3 degradation, but the mechanisms remain still unclear. An E3 ligase that has been implicated in specific IRF3 ubiquitination is Ro52 (TRIM21) [33] which, interestingly has been localized to cytoplasmic bodies or speckles. The recruitment of ubiquitin to peroxisomes has been previously shown, for example peroxisome PTS-receptors are ubiquitinated, which is crucial for their function [17] and peroxisomes themselves can be degraded by autophagy, a process termed pexophagy, a process may be involved here. In summary our work shows that the pestivirus protein N^pro^ disrupts the host innate immune response by associating with IRF3 on mitochondria before relocating together to peroxisomes. There is co-localisation of ubiquitin with peroxisomes containing IRF3, which we speculate may lead to enhanced degradation of IRF3. In targeting IRF3 for enhanced degradation, N^pro^ simultaneously inactivates two crucial host defense pathways of apoptosis and interferon, providing an environment for virus persistence.

## References

[1] Benedict CA, Norris PS, Ware CF (2002) To kill or be killed: viral evasion of apoptosis. Nature Immunology 3: 1013–1018. 12407409 10.1038/ni1102-1013

[2] Taylor KE, Mossman KL (2013) Recent advances in understanding viral evasion of type I interferon. Immunology 138: 190–197. 23173987 10.1111/imm.12038 PMC3573272

[3] Peterhans E, Schweizer M (2010) Pestiviruses: how to outmanoeuvre your hosts. Vet Microbiol 142: 18–25. 19846261 10.1016/j.vetmic.2009.09.038

[4] Moennig V, Plagemann PG (1992) The pestiviruses. Adv Virus Res 41: 53–98. 1315479 10.1016/s0065-3527(08)60035-4

[5] La Rocca SA, Herbert RJ, Crooke H, Drew TW, Wileman TE, et al (2005) Loss of interferon regulatory factor 3 in cells infected with classical swine fever virus involves the N-terminal protease, N^pro^ . J Virol 79: 7239–7247. 15890962 10.1128/JVI.79.11.7239-7247.2005 PMC1112113

[6] Szymanski MR, Fiebach AR, Tratschin JD, Gut M, Ramanujam VM, et al (2009) Zinc binding in pestivirus N(^pro^) is required for interferon regulatory factor 3 interaction and degradation. J Mol Biol 391: 438–449. 19540847 10.1016/j.jmb.2009.06.040

[7] Bauhofer O, Summerfield A, Sakoda Y, Tratschin JD, Hofmann MA, et al (2007) Classical swine fever virus N^pro^ interacts with interferon regulatory factor 3 and induces its proteasomal degradation. J Virol 81: 3087–3096. 17215286 10.1128/JVI.02032-06 PMC1866024

[8] Achmüller C, Kaar W, Ahrer K, Wechner P, Hahn R, et al (2007) N(pro) fusion technology to produce proteins with authentic N termini in E. coli. Nat Methods 4: 1037–1043. 18026112 10.1038/nmeth1116

[9] Killip MJ, Young DF, Gatherer D, Ross CS, Short JA, et al (2013) Deep sequencing analysis of defective genomes of parainfluenza virus 5 and their role in interferon induction. J Virol 87: 4798–4807. 23449801 10.1128/JVI.03383-12 PMC3624313

[10] Vince JE, Tschopp J (2010) IRF-3 partners Bax in a viral-induced dance macabre. EMBO J 29: 1627–1628. 20485298 10.1038/emboj.2010.79 PMC2876974

[11] Seth RB, Sun L, Ea CK, Chen ZJ (2005) Identification and characterization of MAVS, a mitochondrial antiviral signaling protein that activates NF-kappaB and IRF 3. Cell 122: 669–682. 16125763 10.1016/j.cell.2005.08.012

[12] Belgnaoui SM, Paz S, Hiscott J (2011) Orchestrating the interferon antiviral response through the mitochondrial antiviral signaling (MAVS) adapter. Curr Opin Immunol 23: 564–572. 21865020 10.1016/j.coi.2011.08.001

[13] Hou F, Sun L, Zheng H, Skaug B, Jiang QX, et al (2011) MAVS forms functional prion-like aggregates to activate and propagate antiviral innate immune response. Cell 146: 448–461. 21782231 10.1016/j.cell.2011.06.041 PMC3179916

[14] Chattopadhyay S, Marques JT, Yamashita M, Peters KL, Smith K, et al (2010) Viral apoptosis is induced by IRF3-mediated activation of Bax. EMBO J 29: 1762–1772. 20360684 10.1038/emboj.2010.50 PMC2876960

[15] Zemirli N, Arnoult D (2012) Mitochondrial anti-viral immunity. Int J Biochem Cell Biol 44: 1473–1476. 22664327 10.1016/j.biocel.2012.05.018

[16] Dixit E, Boulant S, Zhang Y, Lee AS, Odendall C, et al (2010) Peroxisomes are signaling platforms for antiviral innate immunity. Cell 141: 668–681. 20451243 10.1016/j.cell.2010.04.018 PMC3670185

[17] Platta HW, Erdmann R (2007) Peroxisomal dynamics. Trends in Cell Biology 17: 474–484. 17913497 10.1016/j.tcb.2007.06.009

[18] Hoepfner D, Schildknegt D, Braakman I, Philippsen P, Tabak HF (2005) Contribution of the endoplasmic reticulum to peroxisomal formation. Cell 122: 89–95. 10.1016/j.cell.2005.04.025 16009135

[19] Lloyd RE (2012) How Do Viruses Interact with Stress-Associated RNA Granules? PLoS Pathog 8: e1002741. 22761570 10.1371/journal.ppat.1002741 PMC3386173

[20] Tait SW, de Vries E, Maas C, Keller AM, D'Santos CS, et al (2007) Apoptosis induction by Bid requires unconventional ubiquitination and degradation of its N-terminal fragment. J Cell Biol 179: 1453–1466. 18166654 10.1083/jcb.200707063 PMC2373500

[21] Ruggli N, Summerfield A, Fiebach AR, Guzylack-Piriou L, Bauhofer O, et al (2009) Classical swine fever virus can remain virulent after specific elimination of the interferon regulatory factor 3-degrading function of N^pro^ . J Virol 83: 817–829. 18987150 10.1128/JVI.01509-08 PMC2612357

[22] Renault TT, Manon S (2011) Bax: Addressed to kill. Biochimie 93: 1 379–1391. 10.1016/j.biochi.2011.05.013 21641962

[23] Martinou JC, Youle RJ (2011) Mitochondria in apoptosis: Bcl-2 family members and mitochondrial dynamics. Dev Cell 21: 92–101. 21763611 10.1016/j.devcel.2011.06.017 PMC3156409

[24] Ettema TJ, Huynen MA, de Vos WM, van der Oost J (2003) TRASH: a novel metal-binding domain predicted to be involved in heavy-metal sensing, trafficking and resistance. Trends Biochem Sci 28: 170–173. 12713899 10.1016/S0968-0004(03)00037-9

[25] Castanier C, Arnoult D (2010) Mitochondrial localization of viral proteins as a means to subvert host defense. Biochim Biophys Acta 1813: 575–83. 20807553 10.1016/j.bbamcr.2010.08.009

[26] Sharma S, Fitzgerald KA (2010) Viral defence: it takes two MAVS to Tango. Cell 141: 570–572. 20478250 10.1016/j.cell.2010.04.043

[27] Li Y, Chen R, Zhou Q, Xu Z, Li C, et al (2012) LSm14A is a processing body-associated sensor of viral nucleic acids that initiates cellular antiviral response in the early phase of viral infection. Proc Natl Acad Sci U S A 109: 11770–11775. 22745163 10.1073/pnas.1203405109 PMC3406844

[28] Paz S, Vilasco M, Arguello M, Sun Q, Lacoste J, et al (2009) Ubiquitin-regulated recruitment of I kappaB kinase epsilon to the MAVS interferon signaling adapter Mol Cell Biol. 29: 3401–1226. 10.1128/MCB.00880-08 PMC2698723 19380491

[29] Doceul V, Charleston B, Crooke H, Reid E, Powell PP, et al (2008) The N^pro^ product of classical swine fever virus interacts with I kappa B alpha, the NF-kappaB inhibitor. J Gen Virol 89: 1881–9. 18632959 10.1099/vir.0.83643-0

[30] Lazarow PB (2011) Viruses exploiting peroxisomes. Curr Opin Microbiol 14: 458–469. 21824805 10.1016/j.mib.2011.07.009

[31] Mohan KV, Som I, Atreya CD (2002) Identification of a type 1 peroxisomal targeting signal in a viral protein and demonstration of its targeting to the organelle J Virol. 76: 2543–2547. 10.1128/jvi.76.5.2543-2547.2002 PMC153815 11836432

[32] Wolff T, O’Neill RE, Palese P (1996) Interaction cloning of NS1-I, a human protein that binds to the nonstructural NS1 proteins of influenza A and B viruses. J Virol 70: 5363–5372. 8764047 10.1128/jvi.70.8.5363-5372.1996 PMC190494

[33] Higgs R, Ní Gabhann J, Ben Larbi N, Breen EP, Fitzgerald KA, et al (2008) The E3 ubiquitin ligase Ro52 negatively regulates IFN-beta production post-pathogen recognition by polyubiquitin-mediated degradation of IRF3. J Immunol 181: 1780–1786. 18641315 10.4049/jimmunol.181.3.1780 PMC2824853

